# Comparison of Small-Area Analysis Techniques for Estimating Prevalence by Race

**Published:** 2010-02-15

**Authors:** Melody S Goodman

**Affiliations:** Department of Preventive Medicine, Stony Brook University Medical Center

## Abstract

**Introduction:**

The Behavioral Risk Factor Surveillance System (BRFSS) is commonly used for estimating the prevalence of chronic disease. One limitation of the BRFSS is that valid estimates can only be obtained for states and larger geographic regions. Limited health data are available on the county level and, thus, many have used small-area analysis techniques to estimate the prevalence of disease on the county level using BRFSS data.

**Methods:**

This study compared the validity and precision of 4 small-area analysis techniques for estimating the prevalence of 3 chronic diseases (asthma, diabetes, and hypertension) by race on the county level. County-level reference estimates obtained through local data collection were compared with prevalence estimates produced by direct estimation, synthetic estimation, spatial data smoothing, and regression. Discrepancy statistics used were Pearson and Spearman correlation coefficients, mean square error, mean absolute difference, mean relative absolute difference, and rank statistics.

**Results:**

The regression method produced estimates of the prevalence of chronic disease by race on the county level that had the smallest discrepancies for a large number of counties.

**Conclusion:**

Regression is the preferable method when applying small-area analysis techniques to obtain county-level prevalence estimates of chronic disease by race using a single year of BRFSS data.

## Introduction

The Behavioral Risk Factor Surveillance System (BRFSS) collects uniform, state-specific data on preventive health practices and risk behaviors that are linked to chronic diseases, injuries, and preventable infectious diseases in adults (1). Although BRFSS provides a wealth of information, valid direct estimation of prevalence can only be calculated at state and larger geographic levels because of the structure of the sampling design and weighting scheme (2,3). The lack of data at the local level hinders the ability to evaluate the effectiveness of public health policy, local public health programs, and public health interventions (4).

Several statistical procedures for small-area analysis have been developed to help fill the local data void. Small-area analysis is a statistical procedure that provides a better estimate when the sample size for an area is too small or nonexistent. These approaches, as discussed in Jia et al (5), address the issue of sample size and, therefore, allow for increased precision of estimates.

The most commonly used methods include direct estimation, synthetic estimation, spatial data smoothing, and regression analysis (6). Each has unique advantages and disadvantages. The simplest, direct county-level estimation, is not recommended; when using BRFSS, estimates should only be produced when there are more than 50 respondents in a subgroup. The synthetic method applies statistics for the state to counties based on demographic characteristics. Although estimates are easy to calculate, they often have bias as they tend to cluster around the state average, are heavily influenced by the more densely populated areas within the state, and often do not reflect the actual variation in the patterns of disease among the counties within a state. Spatial data smoothing uses data from neighboring counties to calculate a weighted moving average. Spatial estimates are dependent on the amount of smoothing and the amount of data available to produce estimates. This approach does not perform well for counties near the US border because there are fewer neighbor counties and, thus, less data available to produce estimates. Multivariable regression analysis with area-specific data as predictors has 2 major advantages over the other approaches: the estimates often have only a small amount of bias, and the quality of estimation can be evaluated through model evaluation statistics. Major limitations of this approach, however, are that models are often multi-level and require county-level data, model building is not straightforward and often time consuming, and there is no systematic way to select a final model.

It is unknown which small-area technique produces the most valid and precise results for racial subgroup estimates on the county level, and the validity and precision of the BRFSS for county-level estimation of chronic disease prevalence have not been discussed in the literature. I examine the validity and precision of BRFSS for estimating the prevalence of disease by racial subgroup on the county level (5).

## Methods

I examined the reliability and accuracy of direct estimation, synthetic estimation, spatial smoothing, and regression for small-area analysis. I used each method to compare 2003 BRFSS prevalence estimates with county-level reference estimates of asthma, hypertension, and diabetes for non-Hispanic whites and non-Hispanic blacks.

### County-level reference estimates

County-level reference estimates were obtained for US counties from publicly available county-level data collected in 2003 (eg, data for New York City come from the New York City Department of Health Community Health Assessment) for which the prevalence of asthma, diabetes, or hypertension was available or could be calculated for non-Hispanic whites and blacks. Most of the prevalence estimates by race and county used in this analysis were available on the Internet; other estimates were obtained by contacting state and county departments of health. For some counties, estimates were not available for all 3 diseases or for both racial subgroups; thus, selection bias is possible. In the 2000 US Census, the percentage of non-Hispanic blacks for those counties included in this analysis varied from 1% to 56% (an average of 9% across counties). Likewise, the percentage of non-Hispanic whites varied from 33% to 96% (an average of 77% across counties). Most counties had a mix of urban and rural areas; 65% of the population in these counties live in urban areas. Seven counties were urban and 10 counties were rural. Because of the variety of geographic locations, demographic composition, and mix of rural, urban, and suburban counties for which county-level estimates were obtained, I believe this analysis is generalizable to US counties not included in this analysis that have similar characteristics.

### BRFSS prevalence estimates

Prevalence estimates for asthma were obtained from a sequence of 2 BRFSS questions. Survey participants were first asked, "Have you ever been told by a doctor, nurse, or other health professional that you had asthma?" If the respondents answered yes they were then asked, "Do you still have asthma?" Respondents responding yes to both questions were considered to have asthma. The prevalence of diabetes and hypertension were calculated using survey participants' responses to the questions "Have you ever been told by a doctor that you have diabetes?" and "Have you ever been told by a doctor, nurse, or other health professional that you have high blood pressure?" respectively. Respondents answering yes were then asked, "Was this only when you were pregnant?"; respondents answering "yes, but only during pregnancy" were considered as not having chronic diabetes or hypertension for the purpose of this analysis.

### Estimation Methods

**Direct estimation**

Direct prevalence estimates for asthma, hypertension, and diabetes were calculated by race and county by using weighted 2003 BRFSS data for counties with more than 50 respondents.

**Synthetic estimation**

The synthetic estimate for county *i* is the sum of the 3-way, age-race-sex tabulated rates at the state level for demographic category *j* over all demographic groups, weighted by the proportion of the county population in each demographic category:

Equation 1

p^i=∑jnijnip^j

where ${\hat{p}}_{i}$ is the estimated prevalence of disease in county *i*, *n_ij_* is the number of people in county *i* that belong to demographic group *j*, $n_{i}=\sum_{j}n_{\mathrm{ij}}$ is the total population in county *i*, and ${\hat{p}}_{j}$ is the estimated state level age-race-sex prevalence rates.

The demographic population estimates (*n_ij_* and *n_i._*) are from the 2000 census. The estimated state–level 3-way prevalence rates for asthma, hypertension, and diabetes were calculated by using weighted 2003 BRFSS data.

**Spatial smoothing**

Spatial prevalence estimates were obtained by using the weighted "head-banging" spatial data smoothing algorithm (7). The median estimated prevalence rate for neighboring counties was calculated (*u_i_*). The counties were then grouped according to whether their estimated prevalence rates (${\hat{p}}_{i}$) fell above or below *u_i_*, and these 2 quantities were calculated:

1. high screen for county *i* = weighted median prevalence of neighboring counties ≥*u_i_*
2. low screen for county *i* = weighted median prevalence of neighboring counties <*u_i_*

The weights were based on the county population. If the estimated prevalence rate for county *i* was between the high and low screen, its value was unchanged. However, if ${\hat{p}}_{i}$ was larger than its high screen, then its value was changed to the high screen and if ${\hat{p}}_{i}$ was less then the low screen its value was changed to the low screen.

**Regression**

Multilevel logistic regression models with random effects were used to obtain county prevalence estimates:

Equation 2

logit(p_*ij*_) = *χ*' *β* + *α*_*i*_

where **x**_*ij*_ = (*χ*_*ij*1_,...,*χ_ij_*)' is the vector of *q* covariates, *β* = (*β*_1_,...,*β*_q_)' is the corresponding vector of fixed effects, and α*_i_* is the random effect for county. Demographic variables were added to the model first, followed by county-level socioeconomic characteristics obtained from 2000 census data including poverty rate, median household income, and proportion of adults with less than high school education. The random effect was assumed to be normally distributed with a mean of 0 and a variance equal to σ^2^. If the random effect term was too small to affect the accuracy of estimated county prevalence rates (<0.001%), to simplify analysis, the random effects were not estimated and were assumed to have a value of 0. Even when the random effect term was assumed to be 0 it was still included in the model to improve estimation for the fixed effects and to ensure correct selection of the variables for inclusion in the model (8,9). A final model was selected on the basis of significance of covariates and model fit. Once the regression parameters were calculated, I estimated the county prevalence rates by race.

### Data analysis

Analysis was conducted by using SAS/STAT version 9.1 (SAS Institute, Inc, Cary, North Carolina) with SAS-callable Sudaan version 9.0 (RTI, Research Triangle Park, North Carolina) to adjust for the complex sampling design in BRFSS (10-13). BRFSS county prevalence estimates obtained by using the small-area analysis techniques were validated by comparing them to the county-level reference estimates obtained through local data collection. Counties for which no reference estimate was available were excluded from analysis. Discrepancies between the county-level reference estimates (*c_i_*) and the BRFSS estimated prevalence rates (*p_i_*) were examined by using scatterplots of BRFSS estimates (*p_i_*) versus county level reference estimates (*c_i_*) and discrepancy statistics (5):

1. Pearson and Spearman correlation coefficients
2. Mean square error (MSE): $\frac{1}{N}\sum_{i=1}^{N}{\left(p_{i}-c_{i}\right)}^{2}$
3. Mean absolute difference (MAD): $\frac{1}{N}\sum_{i=1}^{N}|p_{i}-c_{i}|$
4. Mean relative absolute difference (MRAD): $\frac{1}{N}\sum_{i=1}^{N}\frac{|p_{i}-c_{i}|}{c_{i}}$
5. Rank statistics (14): $\frac{\sqrt{12}}{N}\sum_{i=1}^{N}[(\frac{\mathrm{rank}(p_{i}-c_{i})}{N+1}-\frac{1}{2})\times (p_{i}-c_{i})]$

In each equation, *N* is the number of counties (5).

BRFSS does not identify counties with a population of less than 150,000; these counties were excluded from analysis. Pearson correlation coefficient, MSE, MAD, and MRAD are parametric statistics and assume normality in test assumptions. For the purpose of this analysis I assumed normality of the errors between the small-area BRFSS estimates and the county-level reference estimates via the central limit theorem; all discrepancy statistics were based on sample sizes greater than 50. Spearman correlation coefficients and rank statistics are provided as nonparametric alternatives in case the normality assumption is violated.

The Pearson and Spearman correlation statistics are numerical representations of scatterplots and provide a more objective way to test the hypothesis that the BRFSS prevalence estimates and county-level estimates are linearly correlated. Ideally, the small-area BRFSS prevalence estimates (*p_i_*) would be equal to the county-level reference estimates (c_*i*_) and therefore lie on straight line with a 45-degree angle. By using the Pearson correlation coefficient and its nonparametric counterpart the Spearman correlation coefficient, I test the null hypothesis that the BRFSS estimates and reference estimates are not linearly related. Correlation coefficients close to 1 would indicate that BRFSS prevalence estimates and county-level estimates have a high linear correlation, thus the small-area analysis technique produces valid and precise estimates. Good small-area analysis prevalence estimates would have MSE, MAD, MRAD, and rank statistics close to 0, indicating very little discrepancy with county-level estimates.

## Results

Of the 1,937 BRFSS estimates of race by county, 906 (47%) had subgroup sample sizes of less than 50, the minimum needed for direct estimation of prevalence (2). For counties with subgroup sample size more than 50, the average number of respondents per subgroup was 124 (minimum of 51 and maximum of 970).

For the prevalence of asthma by race, 190 BRFSS prevalence estimates were compared with the corresponding 190 county-level reference estimates. Direct estimation produced the largest discrepancy statistics (Table 1). Synthetic estimation showed improvements over direct estimation, producing the largest significant correlation coefficients. Spatial smoothing ranged from second worst (algorithm applied once) to second best (algorithm repeated 20 times). Regression was the best small-area analysis technique for estimating the prevalence of asthma by race at the county level (Table 1); although the correlation coefficients were not significant, they were closest to 1 in magnitude, and the MSE, MAD, MRAD, and rank statistics were closest to 0.

For the prevalence of diabetes by race, 181 county-level reference estimates were compared with the corresponding BRFSS prevalence estimates. Direct estimation had the largest discrepancy. Spatial smoothing ranged from second best to second worst depending on the amount of smoothing (number of times algorithm is repeated). Synthetic estimation performed slightly better than direct estimation and produced significant correlation coefficients. Regression showed significance only in the nonparametric Spearman correlation coefficient. Overall, the regression approach showed the least amount of discrepancy, making it the better small-area analysis technique for estimating the prevalence of diabetes by race on the county level (Table 2).

For the prevalence of hypertension by race, 182 county-level reference estimates were compared with BRFSS estimates. Direct estimation and spatial smoothing showed the biggest discrepancies (Table 3). Even when the amount of smoothing was increased (algorithm repeated >20 times), this technique consistently displayed large discrepancies. Synthetic estimation showed improvements over direct estimation and spatial smoothing. Regression showed marginal improvements over synthetic estimation.

## Discussion

I examined data for non-Hispanic whites and non-Hispanic blacks because the prevalence of asthma, hypertension, and diabetes were consistently measured for these groups. Other racial/ethnic groups for which reference prevalence estimates are consistently measured were hard to obtain because of the small sample size (eg, Asians, Native Americans, Pacific Islanders, Hispanics). Generalizability of small-area analysis techniques for these subpopulations has not been validated and is an area for future research.

Direct estimation had the largest discrepancies, likely because the BRFSS is not designed to produce subpopulation county-level estimates because of small subgroup sample sizes on the county level. This was especially true for non-Hispanic blacks and demonstrates a major limitation of this technique. Although regression appears to be the best small-area analysis technique, synthetic estimation and spatial smoothing often performed better than regression when no county-level variables were significantly associated with the outcome. Other smoothing methods may be appropriate for this type of analysis, which raises questions about the proper choice of smoothing technique and choosing the appropriate degree of smoothing for estimation. The synthetic method has been used widely in public health practice, likely because of the ease of calculation. However, researchers have also used Bayesian methods and complex regression analysis to produce estimates; a comparison of these approaches may also prove beneficial.

This area of research is limited by the lack of systematic local data collection of chronic disease prevalence by race/ethnicity. Development and refinement of small-area analysis techniques relies heavily on statistically sound reference estimates. It was challenging to obtain county-level reference estimates by race; this was especially true for non-Hispanic blacks as the estimates were often unstable because of small sample sizes. There is a potential for selection bias based on publicly available data used as reference estimates.

Statistically sound local-level estimates of chronic disease by race may improve our ability to address racial/ethnic disparities in chronic disease using evidence-based public health. Small-area analysis can provide reliable county-level estimates for the prevalence of chronic disease by race using BRFSS data when a county has few respondents. BRFSS data is a probability sample of US households with a telephone. Telephone coverage varies by state and subpopulation, which raises issues of selection bias in BRFSS data collection. Despite its limitations, BRFSS remains the best available health data for substate estimation.

## Figures and Tables

**Table 1:** Discrepancy Statistics Comparing 2003 Behavioral Risk Factor Surveillance System (BRFSS) Estimates and 2003 County-Level Estimates for Prevalence of Asthma^a^

**Discrepancy Statistics**	Direct	Spatial Smoothing	Synthetic	Regression
Pearson correlation coefficient	0.0250	−0.0272	0.7624	0.8277
Spearman correlation coefficient	−0.0413	0.0394	0.6820	0.7721
Mean square error (MSE)	0.2768	0.3044	0.1529	0.1496
Mean absolute difference (MAD)	0.4443	0.4688	0.3557	0.3451
Mean relative absolute difference (MRAD)	3.9961	3.8929	0.6481	0.5278
Rank statistics	0.3141	0.3463	0.2372	0.1471

a Correlation coefficients close to 1 indicate that BRFSS prevalence estimates and county-level estimates have a high linear correlation, thus producing valid and precise estimates. MSE, MAD, MRAD, and rank statistics close to 0 indicate little discrepancy with county-level estimates.

**Table 2:** Discrepancy Statistics Comparing 2003 Behavioral Risk Factor Surveillance System (BRFSS) Estimates and 2003 County-Level Estimates for Prevalence of Diabetes^a^

**Discrepancy Statistics**	Direct	Spatial Smoothing	Synthetic	Regression
Pearson correlation coefficient	0.0515	0.1096	0.0541	0.1328
Spearman correlation coefficient	0.1291	0.1506	0.2068	0.2309
Mean square error (MSE)	0.0121	0.0527	0.0083	0.0020
Mean absolute difference (MAD)	0.0655	0.2396	0.0563	0.0351
Mean relative absolute difference (MRAD)	0.8819	2.0876	0.6075	0.5554
Rank statistics	0.0872	0.1622	0.0688	0.0178

a Correlation coefficients close to 1 indicate that BRFSS prevalence estimates and county-level estimates have a high linear correlation, thus producing valid and precise estimates. MSE, MAD, MRAD, and rank statistics close to 0 indicate little discrepancy with county-level estimates.

**Table 3:** Discrepancy Statistics Comparing 2003 Behavioral Risk Factor Surveillance System (BRFSS) Estimates and 2003 County-Level Estimates for Prevalence of Hypertension^a^

**Discrepancy Statistics**	Direct	Spatial Smoothing	Synthetic	Regression
Pearson correlation coefficient	0.0599	0.1984	−0.0525	0.0573
Spearman correlation coefficient	0.0913	0.1294	−0.0731	0.2153
Mean square error (MSE)	0.0466	0.1046	0.0386	0.0315
Mean absolute difference (MAD)	0.1382	0.2396	0.1987	0.1654
Mean relative absolute difference (MRAD)	0.4809	0.8327	0.2720	0.2067
Rank statistics	0.1805	0.2601	0.0965	0.0535

a Correlation coefficients close to 1 indicate that BRFSS prevalence estimates and county-level estimates have a high linear correlation, thus producing valid and precise estimates. MSE, MAD, MRAD, and rank statistics close to 0 indicate little discrepancy with county-level estimates.
